# The predictive value of the ultrasound grayscale ratio for identifying malignant thyroid nodules

**DOI:** 10.3389/fendo.2025.1665113

**Published:** 2025-08-29

**Authors:** Kaimei Lian, Teng Lin

**Affiliations:** Department of Ultrasound, The First Affiliated Hospital of Shantou University Medical College, Shantou, China

**Keywords:** thyroid nodule, Chinese thyroid imaging reporting and data system, ultrasound grayscale ratio, malignant thyroid nodule, imageJ

## Abstract

**Purpose:**

To assess the clinical utility of ultrasound grayscale ratio (UGSR) in distinguishing between benign and malignant thyroid nodules.

**Methods:**

We conducted a retrospective analysis of patients diagnosed with thyroid nodules between January 2017 and December 2021. Malignancy and benignity were determined based on histopathology (biopsy or surgery) as the reference standard. Conventional ultrasonography (US) was performed to measure the maximum diameter of each nodule and assess positive features of the Chinese Thyroid Imaging Reporting and Data System (C-TIRADS). UGSR was calculated as the ratio of nodule grayscale value to surrounding thyroid parenchyma grayscale value, quantified using ImageJ software. Two experienced radiologists used ImageJ software for UGSR measurements. Logistic regression analysis examined the association between UGSR and thyroid malignancy. A receiver operating characteristic (ROC) curve analysis was conducted, and the area under the curve (AUC) was calculated to assess UGSR’s effectiveness in distinguishing between benign and malignant thyroid nodules. The UGSR cut-off value was established through ROC curve analysis.

**Results:**

A total of 125 nodules (78 benign, 47 malignant) were evaluated. Significant differences were observed between benign and malignant nodules in maximum diameter (*p* = 0.042), UGSR (*p* < 0.001), and C-TIRADS features (*p* < 0.001), supporting UGSR’s diagnostic utility. However, no significant intergroup differences were observed in gender or age distribution (*p*>0.05 for both). Multivariable logistic regression analysis identified UGSR, irregular margins, taller-than-wide orientation, and microcalcifications as independent predictive factors for differentiating malignant from benign thyroid nodules (all *p*<0.05). The diagnostic performance evaluation demonstrated that UGSR achieved an AUC of 0.852 (95% CI: 0.792 - 0.912), with a sensitivity of 63.83% and specificity of 92.31%. UGSR showed significantly superior diagnostic accuracy compared to markedly hypoechogenicity (*p*<0.05).

**Conclusion:**

UGSR demonstrated high specificity (92.31%) and reliability in differentiating malignant from benign thyroid nodules, suggesting its potential as a quantitative adjunct to ultrasound diagnosis, though sensitivity (63.83%) warrants combination with other features.

## 1 Introduction

Thyroid nodules are a widespread endocrine condition. While most thyroid nodules are benign, 5 - 10% are malignant (1). The high prevalence of this condition highlights the importance of effective screening and clinical management of thyroid nodules. Thyroid nodule detection rates vary by age and gender, with higher rates observed in women (2). Ultrasound (US) is the preferred method for assessing thyroid nodules as it offers crucial information on their size, shape, and morphological features, which aid in determining whether they are benign or malignant (3–6). Chinese Thyroid Imaging Reporting and Data System (C-TIRADS) stratifies nodules by malignancy risk using sonographic features (e.g., echogenicity, margins) (7).

The evaluation of thyroid nodules typically begins with US as the first-line imaging modality. On ultrasounds, the echogenic characteristics of thyroid nodules help differentiate benign from malignant lesions. Based on their echogenicity, a nodule may be classified as anechoic, markedly hypoechogenic, hypoechogenic, isoechogenic, or hyperechogenic. These classifications assist physicians in making initial diagnoses and determining the need for subsequent fine needle aspiration biopsies (FNA). Previous studies have shown that hypoechogenicity and markedly hypoechogenicity nodules are more frequently associated with malignancy, while isoechogenicity and hyperechogenicity nodules are typically benign (8, 9). However, these echogenic features lack specificity and often fail to meet clinical requirements. Although the link between thyroid nodule echogenicity and cancer risk has been established, it remains unclear in certain cases.

In clinical practice, evaluating thyroid nodules via ultrasonography often relies on subjective judgment, leading to inconsistent or ambiguous outcomes. Previous studies have reported significant interobserver variability in the interpretation of ultrasound images, which may contribute to unnecessary diagnostic or treatment interventions for thyroid nodules (10). To address this, the ultrasound grayscale ratio (UGSR) quantified by ImageJ software offers a potential solution by replacing visual interpretation with a quantitative measurement. This approach could provide greater objectivity and improved interobserver consistency, making it a reliable alternative for echogenicity assessment (11). Since different ultrasound machine parameters may affect raw grayscale values, we used UGSR instead of raw grayscale values for standardization.

This study aims to enhance diagnostic accuracy by quantitatively analyzing thyroid nodule echogenicity and investigating its association with benign and malignant pathology.

## 2 Materials and methods

### 2.1 Study population

We reviewed patients treated for thyroid nodules from January 2017 to December 2021. Malignancy and benignity were determined based on histopathology (biopsy or surgery) as the reference standard. Inclusion criteria: (1) thyroid ultrasound within a month before biopsy or surgery; (2) had a thyroid nodule biopsy or surgery at our hospital; (3) the ultrasound images and thyroid nodule pathology were clear. Exclusion Criteria: (1) ultrasound images inadequately displayed the thyroid nodule and its surrounding tissue; (2) nodules <5 mm or >40 mm; (3) cystic or calcified nodules with unclear borders; (4) significantly changed thyroid parenchyma echogenicity or Hashimoto’s thyroiditis; (5) previous thyroid biopsy, surgery, or neck radiation therapy. This cross-sectional study analyzed retrospective data from patients who underwent thyroid ultrasound and subsequent histopathology.

The protocol of this retrospective study has been reviewed and approved by the Ethics Review Committee of the First Affiliated Hospital of Shantou University Medical College (Ethics Review No.: B - 2024 - 239), and the committee waived the requirement for informed consent.

### 2.2 Instruments and methods

Our study used the Siemens Acuson S2000 ultrasound machine’s 4 – 9 MHz linear transducer. The patient lay supine with an extended head, exposing the neck to scan the nodule’s location, shape, borders, echogenicity, calcifications, and internal structures, and measure its maximum diameter. A radiologist acquired all images with 10 years of experience in thyroid imaging. Two experienced radiologists analyzed images of thyroid nodules, scoring features based on C-TIRADS criteria. Each positive feature—vertical position, solidity, very hypoechoicity, microcalcification, and margin blurring—received one point, while comet-tail artifacts resulted in a one-point deduction. The total score was calculated as follows: Category 1 for no nodule (0% malignancy rate), category 2 for a score of -1 (0% malignancy rate), category 3 for a score of 0 (<2% malignancy rate), category 4a for a score of 1 (2%-10% malignancy rate), category 4b for a score of 2 (>10%-50% malignancy rate), category 4c for scores of 3 to 4 (>50%-90% malignancy rate), and category 5 for a score of 5 (>90% malignancy rate) (7). The two radiologists were unaware of the pathology results. If two radiologists disagree on an image feature’s definition and diagnosis, the final decision will be based on their consensus.

**Figure 1:**
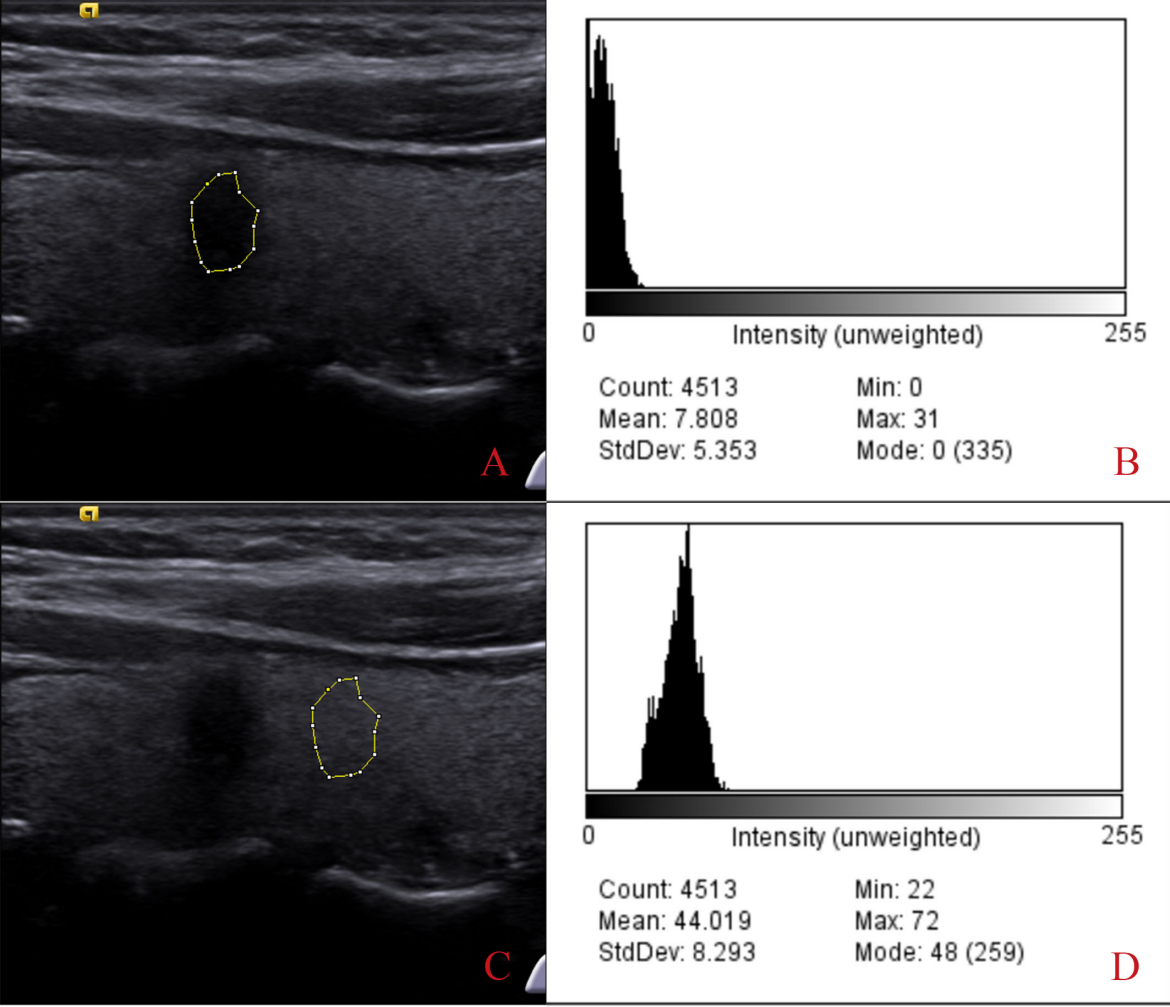
Process of extracting the grayscale ratio from thyroid nodules. Images **(A, B)** show the extracted gray value of the thyroid nodule, which is 7.808. Images **(C, D)** show the gray values extracted from the adjacent thyroid tissue (44.019). The ultrasound grayscale ratio (UGSR) is 7.808/44.019 = 17.74%.

Two experienced radiologists used ImageJ software for UGSR measurements. Two radiologists with 10 years of thyroid imaging experience performed all measurements, blinded to pathology results. ImageJ software measured the grayscale value of the entire nodule and surrounding thyroid tissue, ensuring consistent sampling depth (8, 12). The measurement area for the surrounding tissue matched the nodule’s size. Perform five measurements for each and average them. UGSR was calculated as the ratio of nodule grayscale value to surrounding thyroid parenchyma grayscale value, quantified using ImageJ software (**Figure 1**). ROIs were measured at the same depth in transverse/longitudinal planes. We sampled the entire nodule for nodules with heterogeneous echogenicity to avoid bias. A total of 125 nodules (78 benign, 47 malignant) were evaluated.

Thirty randomly selected nodules were measured twice by the same radiologist to assess intra-observer reproducibility (ICC).

### 2.3 Statistical methods

All statistical analyses were performed using SPSS software (version 26.0; IBM Corp., Armonk, NY, USA) and MedCalc Statistical Software (version 18.0; MedCalc Software Ltd, Ostend, Belgium). Continuous variables were expressed as mean ± standard deviation (SD) for normally distributed data or median with interquartile range (IQR) for non-normally distributed data. Categorical variables were presented as frequencies and percentages. Comparative analyses were conducted using: Independent samples *t*-test or Mann-Whitney *U* test for continuous variables; Chi-square test or Fisher’s exact test for categorical variables; One-way ANOVA to compare UGSR distributions across different C-TIRADS categories, with *post-hoc* correlation analysis. For logistic regression modeling, we adopted a focused approach: Each model incorporated either UGSR or marked hypoechogenicity as the primary sonographic variable; Four additional covariates were consistently included: maximum diameter, margin characteristics, taller-than-wide orientation, and microcalcifications; Nodule malignancy status served as the dichotomous dependent variable. Diagnostic performance was evaluated through receiver operating characteristic (ROC) curve analysis, calculation of the area under the curve (AUC) with 95% confidence intervals (CI), and sensitivity and specificity determinations at optimal cut-off values. The UGSR cut-off value was established through ROC curve analysis. A two-tailed *p*-value <0.05 was considered statistically significant for all analyses.

## 3 Results

This retrospective study encompassed 118 patients, accounting for 125 nodules. Among these, 78 nodules were classified as benign, comprising 66 nodular goiters and 12 follicular adenomas. The remaining 47 nodules were categorized as malignant, including 46 papillary carcinomas and one follicular adenocarcinoma, as detailed in **Table 1**. In the cohort with benign nodules, the mean age was 46.79 ± 13.89 years, and the maximum nodule diameter was 20.05 mm (range: 14.10 – 26.30 mm). Conversely, in the cohort with malignant nodules, the mean age was 45.19 ± 13.73 years, with a maximum nodule diameter of 12.30 mm (range: 7.85 – 15.08 mm). Significant differences were observed between benign and malignant nodules in maximum diameter (*p* = 0.042), UGSR (*p* < 0.001), and C-TIRADS features (*p* < 0.001), supporting UGSR’s diagnostic utility. Conversely, the differences between the two groups concerning gender and age did not reach statistical significance (refer to **Table 2**). Despite a higher proportion of females in both benign (85.9%) and malignant (89.4%) groups, no significant gender difference was observed (*p* = 0.574), likely due to matched recruitment by nodule size/suspicion rather than population prevalence. The *ICC* for the measurement of thyroid nodule UGSR was 0.964 (*p* < 0.001), demonstrating a high level of reproducibility. Furthermore, the correlation between UGSR and the C-TIRADS was 0.540 (*p* < 0.001), as presented in **Table 3**.

**Table 1:** Pathologic classification of thyroid nodules.

Pathology results	Number of thyroid nodules (%)
Nodular goiter	66 (52.8)
Follicular thyroid adenoma	12 (9.6)
Papillary thyroid carcinoma	46 (36.8)
Follicular thyroid carcinoma	1 (0.8)
Total	125

Data are the number of thyroid nodules, and numbers in parentheses are percentages.

**Table 2:** Clinical and ultrasound characteristics of patients with thyroid nodules.

Variable	Benign (n = 78)	Malignant (n = 47)	*p*
Gender			0.574^a^
Male	11	5	
Female	67	42	
Age (year), mean ± SD	46.79 ± 13.89	45.19 ± 13.73	0.531^c^
Tumor size (mm), median (IQR)	20.05 (14.10 - 26.30)	12.30 (7.85 - 15.08)	0.042^c^
Margin (Ill-defined/irregular or extrathyroidal extension)			<0.001^a^
Yes	5	36	
No	73	11	
Vertical orientation			<0.001^a^
Yes	4	23	
No	74	24	
Microcalcifications			<0.001^a^
Yes	4	27	
No	74	20	
Markedly hypoechoic			<0.001^a^
Yes	3	12	
No	75	35	
C-TIRADS			<0.001^b^
4a	71	1	
4b	3	13	
4c	3	30	
5	1	3	
UGSR (%), median (IQR)	82.37 (52.63 - 100.52)	39.26 (29.92 - 53.58)	<0.001^d^

SD, Standard deviation; IQR, Interquartile range; C-TIRADS, Chinese thyroid imaging reports and data systems; UGSR, Ultrasound grayscale ratio.

^a^Determined with the Chi-square test; ^b^Determined with the Fisher’s exact test;

^c^Determined with the *t*-test; ^d^Determined with the Mann-Whitney *U* test.

**Table 3:** Distribution of UGSR of thyroid nodule in C-TIRADS 4 and 5.

C-TIRAD category	Number (%)	UGSR (IQR)
4a	72 (57.6)	83.51 (55.83 - 105.23)
4b	16 (12.8)	45.92 (36.23 - 68.07)
4c	33 (26.4)	38.10 (29.80 - 52.54)
5	4 (3.2)	22.87 (13.54 - 33.00)
Total	125 (100)	

Data in parentheses in the left column are percentages unless otherwise indicated; data in the right column are medians with interquartile ranges in parentheses. UGSR, Ultrasound grayscale ratio; IQR, Interquartile range; C-TIRADS, Chinese thyroid imaging reports and data systems.

**Figure 2:**
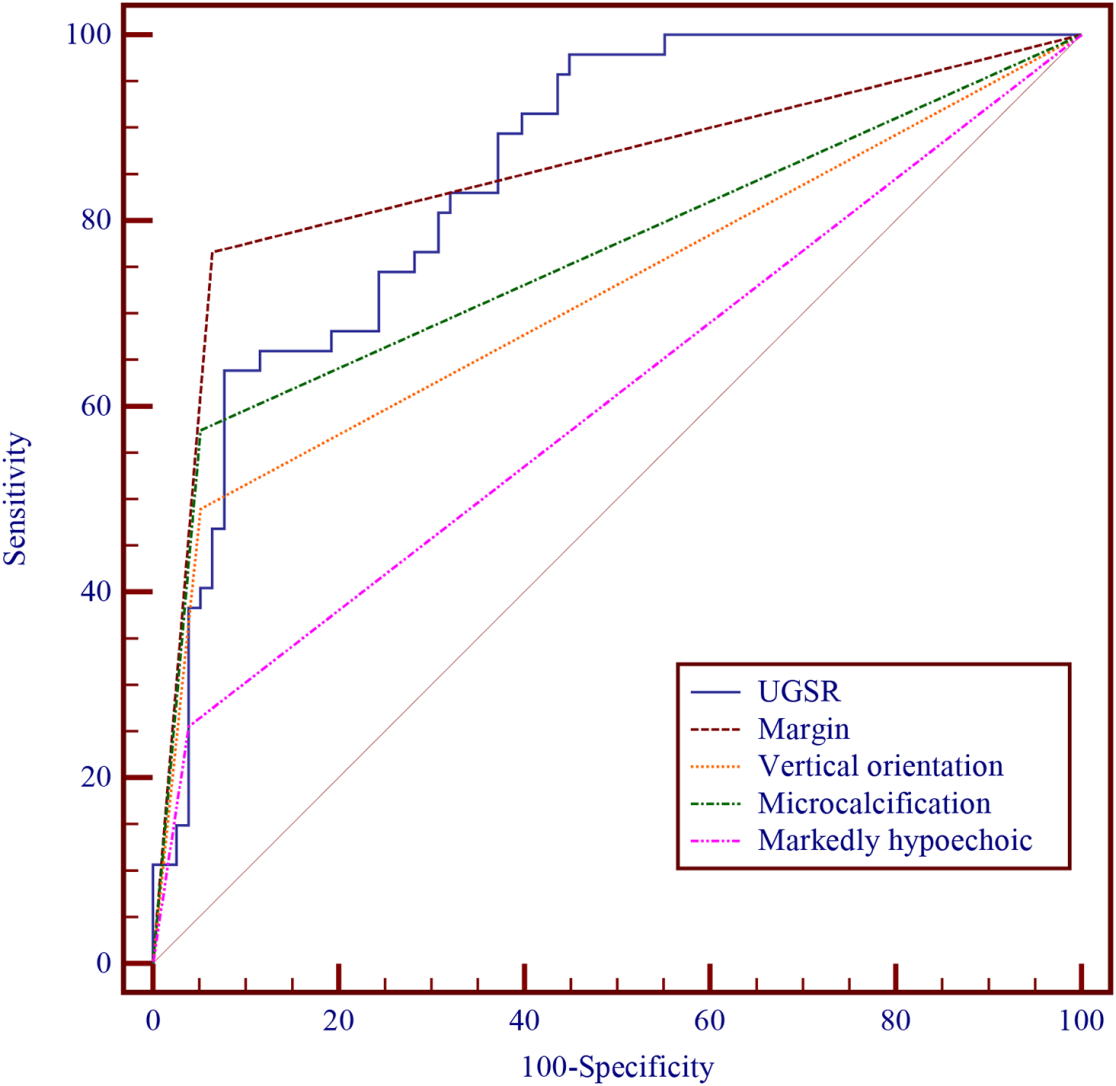
Diagnostic performances of UGSR, margin, vertical orientation, microcalcification, and markedly hypoechoic.

In the logistic regression analysis, we constructed comprehensive models incorporating maximum diameter, margin characteristics, taller-than-wide orientation, microcalcifications, and one sonographic variable (UGSR or marked hypoechogenicity). **Table 4** (Model 1 and Model 2) demonstrated that UGSR emerged as an independent predictive factor for thyroid malignancy (OR: 0.966, 95% CI: 0.936 - 0.997, *p* = 0.033). The diagnostic performance evaluation revealed: UGSR achieved an AUC of 0.852 (95% CI: 0.786 - 0.917) for malignancy discrimination; The optimal predictive probability cut-off was ≤45.027; Sensitivity: 63.83%; Specificity: 92.31%. Comparative analysis demonstrated UGSR’s statistically superior diagnostic accuracy over conventional marked hypoechogenicity (*p*<0.05 by DeLong’s test), with detailed performance metrics in **Table 5** and corresponding ROC curves illustrated in **Figure 2**.

**Table 4:** Logistic regression analysis of different thyroid nodule ultrasound characteristics.

	OR (95%CI)	*p*
Model 1		
Tumor size	1.003 (0.953 – 1.055)	0.921
Margin	18.137 (3.909 – 84.153)	<0.001
Vertical orientation	8.402 (1.596 – 44.221)	0.012
Microcalcifications	12.998 (2.986 – 56.590)	0.001
Markedly hypoechoic	0.420 (0.046 – 3.829)	0.442
Model 2		
Tumor size	1.016 (0.971 – 1.064)	0.494
Margin	6.190 (1.428 – 26.838)	0.015
Vertical orientation	5.773 (1.188 – 28.045)	0.030
Microcalcifications	11.568 (2.625 – 50.971)	0.001
UGSR	0.966 (0.936 – 0.997)	0.033

UGSR, Ultrasound grayscale Ratio; CI, Confidence intervals; OR, Odds ratio.

**Table 5:** Diagnostic performances of UGSR, margin, vertical orientation, microcalcification, and markedly hypoechoic.

Data	Cut-off value	SEN (%)	SPE (%)	AUC (*95%CI*)
UGSR	≤45.027	63.83 (30/47)	92.31 (72/78)	0.852^ce^ (0.786 - 0.917)
Margin	NA	76.60 (36/47)	93.59 (73/78)	0.851^ce^ (0.784 - 0.918)
Vertical orientation	NA	48.94 (23/47)	94.87 (74/78)	0.719^abe^ (0.643 - 0.795)
Microcalcification	NA	57.45 (27/47)	94.87 (74/78)	0.762^e^ (0.686 - 0.837)
Markedly hypoechoic	NA	25.53 (12/47)	96.15 (75/78)	0.608^abcd^ (0.542 - 0.675)

UGSR, Ultrasound grayscale ratio; SEN, Sensitivity; SPE, Specificity; AUC, area under the curve; CI, Confidence intervals.

^a^Compared with the UGSR, *p*<0.05; ^b^Compared with the margin, *p*<0.05; ^c^Compared with the vertical orientation, *p*<0.05; ^d^Compared with the microcalcification, *p*<0.05; ^e^Compared with the markedly hypoechoic, *p*<0.05.

## 4 Discussion

The US is the preferred method for thyroid nodule screening, and the TI-RADS system is increasingly used to standardize nodule grading based on sonographic features. Previous studies have demonstrated that positive C-TIRADS features effectively distinguish between benign and malignant thyroid nodules (7, 13–16). Nevertheless, the accuracy of radiologists in identifying these C-TIRADS features is significantly influenced by their experience and the resolution of the ultrasound equipment. Specifically, assessing nodule echogenicity is particularly vulnerable to subjective interpretation. Prior research has reported limited specificity for diagnosing malignant nodules using hypoechoic echogenicity characteristics when employing hypoechoic features (17, 18).

Additional biopsy procedures are necessary to evaluate further nodules that cannot be definitively classified as benign or malignant. Although FNA is a valuable diagnostic tool, it has several limitations (19), such as challenges related to the puncture procedure and potential inaccuracies due to sampling variability. Additionally, FNA has difficulties in differentiating between follicular adenomas and follicular carcinomas. Enhancing the diagnostic accuracy of thyroid nodules using conventional US and reducing unnecessary FNAs are of significant clinical importance. Therefore, quantifying nodule echogenicity could improve the efficacy of their diagnosis. Since the gain adjustment affects the image’s overall value, direct grayscale-based diagnosis is unreliable. Consequently, this study employed the grayscale ratio to represent nodule characteristics objectively. Since the calcified areas tend to overestimate the grayscale ratio and cystic components underestimate it, only solid nodules without significant calcification were included.

The findings demonstrated that the UGSR of benign nodules was significantly higher than malignant nodules (*p* < 0.001). Tissue echogenicity in ultrasound depends on the number of acoustic interfaces and the degree of acoustic impedance difference between adjacent media. Malignant thyroid nodules primarily consist of cancerous cells, microscopically resulting in a marked reduction of thyroid follicular components. Consequently, this structure does not easily establish an acoustic interface, leading to hypoechoic echogenicity (20). In contrast, thyroid adenomas are benign neoplasms composed of follicular epithelium. Histologically, they contain densely packed follicles in a gelatinous matrix, resulting in homogeneous iso- to hyperechoic patterns. Nodular goiter differs pathologically, featuring focal hyperplasia, small follicles, and areas of epithelial changes, often appearing as uneven or honeycomb-like iso- to hyperechoic lesions (21).

In nodules with heterogeneous echogenicity, manual selection of the sampling frame may introduce bias. Thus, this study used the overall gray value for analysis. The *ICC* for the UGSR measurement of thyroid nodules was 0.964 (*p* < 0.001), confirming high reproducibility and reliability. Our results suggest that UGSR measurements are consistent across different technical parameters, including gain, dynamic range, and transducer frequency. Furthermore, the grayscale ratio normalizes operator-dependent variability, providing an objective quantitative method.

Logistic regression analyses incorporated five sonographic parameters: maximum diameter, margin irregularity, taller-than-wide orientation, microcalcifications, and UGSR or marked hypoechogenicity. As shown in **Table 4** (Models 1 and 2), UGSR was an independent predictor of thyroid malignancy (OR: 0.966; 95% CI: 0.936 - 0.997; *p* = 0.033). Diagnostic performance analysis showed: Superior discriminatory AUC for UGSR (0.852; 95% CI: 0.786 - 0.917); Optimal threshold: ≤45.027; Sensitivity: 63.83%; Specificity: 92.31%; Significantly better than marked hypoechogenicity (*p*<0.05 by DeLong’s test). Our UGSR results (AUC = 0.852) align with Yun et al.’s findings (AUC = 0.856–0.875), supporting its generalizability. However, their cohort achieved higher sensitivity (78.9%–86.9% *vs*. 63.8%), likely because they focused on 3 – 10 mm nodules (8).

Smaller malignant nodule size (12.3 *vs*. 20.1 mm, *p* = 0.042) reflects clinical practice: (1) Suspicious features (e.g., microcalcifications) prompt early intervention; (2) Large benign nodules are often resected for compressive symptoms, introducing selection bias.

The limitations of this study are as follows: (1) Single-center retrospective design, which may introduce selection bias and limit generalizability. Multicenter prospective studies are needed to validate UGSR. (2) Small sample size; future studies should expand cohorts for robustness. While our sample (n=125) suffices for pilot validation (power=0.8, effect size=0.5), multicenter studies with >500 nodules are needed for definitive cut-off establishment. (3) Potential variability across radiologists and ultrasound instruments requires further investigation. (4) Inclusion bias: Only biopsied/surgical nodules were analyzed, which tend to have higher malignancy rates and larger sizes than screening-detected nodules. (5) Reference standard bias: Biopsy/surgical histopathology may miss some benign lesions. (6) Technical variability: UGSR performance may differ with ultrasound systems; protocol standardization is needed. (7) Lack of subgroup analysis by nodule size due to sample size constraints.

## 5 Conclusion

UGSR demonstrated high specificity (92.31%) and reliability in differentiating malignant from benign thyroid nodules, suggesting its potential as a quantitative adjunct to ultrasound diagnosis, though sensitivity (63.83%) warrants combination with other features.

## Data Availability

The raw data supporting the conclusions of this article will be made available by the authors, without undue reservation.

## References

[1] Jiang L Liu D Long L Chen J Lan X Zhang J. Dual-source dual-energy computed tomography-derived quantitative parameters combined with machine learning for the differential diagnosis of benign and Malignant thyroid nodules. Quant Imaging Med Surg. (2022) 12:967–78. doi: 10.21037/qims-21-501, PMID: 35111598 PMC8739151

[2] Li X Du H Luo J Ding W Lai B He J. Comparison of the clinical validity of droplet digital PCR to ARMS-PCR for BRAF V600E mutation detection in thyroid nodules. J Clin Lab Anal. (2020) 34:e23458. doi: 10.1002/jcla.23458, PMID: 32671901 PMC7676211

[3] Min PH Jihye L Young KJ Youngjean PV Miribi R Minah L. Using ultrasonographic features to predict the outcomes of patients with small papillary thyroid carcinomas: a retrospective study implementing the 2015 ATA patterns and ACR TI-RADS categories. Ultrasonography. (2022) 41:298–306. doi: 10.14366/usg.21097, PMID: 34674455 PMC8942744

[4] Zhang WB Xu W Fu WJ He BL Liu H Deng WF. Comparison of ACR TI-RADS, Kwak TI-RADS, ATA guidelines and KTA/KSThR guidelines in combination with SWE in the diagnosis of thyroid nodules. Clin Hemorheol Microcirc. (2021) 78:163–74. doi: 10.3233/CH-201021, PMID: 33579829

[5] Wang Y Dong T Nie F Wang G Liu T Niu Q. Contrast-enhanced ultrasound in the differential diagnosis and risk stratification of ACR TI-RADS category 4 and 5 thyroid nodules with non-hypovascular. Front Oncol. (2021) 11:662273. doi: 10.3389/fonc.2021.662273, PMID: 34123819 PMC8189148

[6] Trimboli P Castellana M Piccardo A Romanelli F Grani G Giovanella L. The ultrasound risk stratification systems for thyroid nodule have been evaluated against papillary carcinoma. A meta-analysis. Rev Endocr Metab Disord. (2021) 22:453–60. doi: 10.1007/s11154-020-09592-3, PMID: 32959174 PMC8087557

[7] Zhou J Yin L Wei X Zhang S Song Y Luo B. 2020 Chinese guidelines for ultrasound Malignancy risk stratification of thyroid nodules: the C-TIRADS. Endocrine. (2020) 70:256–79. doi: 10.1007/s12020-020-02441-y, PMID: 32827126

[8] Gong Y Yao X Yu L Wei P Han Z Fang J. Ultrasound grayscale ratio: a reliable parameter for differentiating between papillary thyroid microcarcinoma and micronodular goiter. BMC Endocr Disord. (2022) 22:75. doi: 10.1186/s12902-022-00994-9, PMID: 35331216 PMC8952271

[9] Low G Bara M Du Y Katlariwala P Croutze R Resch K. Tips for improving consistency of thyroid nodule interpretation with ACR TI-RADS. J Ultrason. (2022) 22:e51–6. doi: 10.15557/JoU.2022.0009, PMID: 35449702 PMC9009349

[10] Ma W Li X Zou L Fan C Wu M. Symmetrical awareness network for cross-site ultrasound thyroid nodule segmentation. Front Public Health. (2023) 11:1055815. doi: 10.3389/fpubh.2023.1055815, PMID: 36969643 PMC10031019

[11] Wu MH Chen KY Chen A Chen CN. Software-based analysis of the taller-than-wide feature of high-risk thyroid nodules. Ann Surg Oncol. (2021) 28:4347–57. doi: 10.1245/s10434-020-09463-w, PMID: 33393024

[12] Hu T Zhou T Zhang Y Zhou L Huang X Cai Y. The predictive value of the thyroid nodule benign and Malignant based on the ultrasound nodule-to-muscle grayscale ratio. J Clin Ultrasound. (2024) 52:51–8. doi: 10.1002/jcu.23601, PMID: 37915163

[13] Hu Y Xu S Zhan W. Diagnostic performance of C-TIRADS in Malignancy risk stratification of thyroid nodules: A systematic review and meta-analysis. Front Endocrinol (Lausanne). (2022) 13:938961. doi: 10.3389/fendo.2022.938961, PMID: 36157473 PMC9492922

[14] Renxu Li Zhenwei L Xiangyu W Luzeng C. Role of echogenic foci in ultrasonographic risk stratification of thyroid nodules: Echogenic focus scoring in the American College of Radiology Thyroid Imaging Reporting and Data System. Front Oncol. (2022) 12:929500. doi: 10.3389/fonc.2022.929500, PMID: 36106124 PMC9465029

[15] Liu J Luo T Zhang H Liu H Gu Y Chen X. Markedly hypoechoic: a new definition improves the diagnostic performance of thyroid ultrasound. Eur Radiol. (2023) 33:7857–65. doi: 10.1007/s00330-023-09828-1, PMID: 37338557

[16] Qu C Li HJ Gao Q Zhang JC Li WM. Alteration trend and overlap analysis of positive features in different-sized benign and Malignant thyroid nodules: based on chinese thyroid imaging reporting and data system. Int J Gen Med. (2024) 17:1887–95. doi: 10.2147/IJGM.S461076, PMID: 38736670 PMC11086651

[17] Prieditis P Radzina M Mikijanska M Liepa M Stepanovs K Grani G. Non-marked hypoechogenic nodules: multicenter study on the thyroid Malignancy risk stratification and accuracy based on TIRADS systems comparison. Medicina (Kaunas). (2022) 58(2):257. doi: 10.3390/medicina58020257, PMID: 35208581 PMC8875125

[18] Petersen M Schenke SA Firla J Croner RS Kreissl MC. Shear wave elastography and thyroid imaging reporting and data system (TIRADS) for the risk stratification of thyroid nodules-results of a prospective study. Diagn (Basel). (2022) 12(1):109. doi: 10.3390/diagnostics12010109, PMID: 35054275 PMC8774661

[19] Fabbri C Fornelli A Fuccio L Giovanelli S Tarantino I Antonini F. High diagnostic adequacy and accuracy of the new 20G procore needle for EUS-guided tissue acquisition: Results of a large multicentre retrospective study. Endosc Ultrasound. (2019) 8:261–8. doi: 10.4103/eus.eus_14_19, PMID: 31115386 PMC6714486

[20] Su Z Bao W Yang G Liu J Zhao B. SOX12 promotes thyroid cancer cell proliferation and invasion by regulating the expression of POU2F1 and POU3F1. Yonsei Med J. (2022) 63:591–600. doi: 10.3349/ymj.2022.63.6.591, PMID: 35619584 PMC9171662

[21] Chang D Wang Q. Diagnostic value of ultrasound elastography and conventional ultrasound for thyroid nodules: a meta-analysis. Quant Imaging Med Surg. (2023) 13:1300–11. doi: 10.21037/qims-22-505, PMID: 36915345 PMC10006152

